# Application of a nursing research order-service model based on a “demand–resource” linkage mechanism

**DOI:** 10.3389/fmed.2026.1770404

**Published:** 2026-03-12

**Authors:** Yanfei Ma, Du Liu, Cheng Yang, Qian Gu, Tianlan Li, Haili Long, Lili Huang, Li Wan

**Affiliations:** 1 Mianyang Central Hospital, School of Medicine, University of Electronic Science and Technology of China, Mianyang, Sichuan, China; 2 Sichuan Institute of Industrial Technology, Deyang, Sichuan, China

**Keywords:** demand–resource theory, discipline development, nurse, nursing, research

## Abstract

**Objective:**

To explore the application effects of a nursing research order service model based on the “demand–resource” linkage in clinical nursing research management, and to provide practical reference for improving the scientific research capacity of nursing staff.

**Methods:**

From May to June 2025, clinical nurses and nursing postgraduate students who participated in the service model were selected as the research subjects. A general information questionnaire, Nursing Research Capacity Self-Assessment Scale, Nursing Research Self-Efficacy Questionnaire, and Organizational Climate for Innovation Scale were used to evaluate participants before and after the intervention. Data were analyzed using SPSS 25.0 software.

**Results:**

A total of 136 research demand orders from clinical nurses were received; after evaluation, 7 were declined and 129 were fulfilled. During the service process, 36 papers and 25 research projects were completed. Among the 126 nurses who submitted research orders, the total score of self-assessed research competence significantly improved after participation (mean difference = −0.611, 95% CI −0.931 to −0.291, *p* < 0.001). Significant improvements were also observed in several subscales: problem identification (mean difference = −0.079, 95% CI −0.148 to −0.010, *p* = 0.025), literature review (mean difference = −0.087, 95% CI −0.150 to −0.024, *p* = 0.007), research design (mean difference = −0.095, 95% CI −0.186 to −0.005, *p* = 0.039), research practice (mean difference = −0.079, 95% CI −0.145 to −0.014, *p* = 0.018), and data processing ability (mean difference = −0.175, 95% CI −0.277 to −0.072, *p* = 0.001). The problem formulation dimension of research self-efficacy also showed a significant increase (mean difference = −0.079, 95% CI −0.145 to −0.014, *p* = 0.018). Among the 34 postgraduate nursing students who provided research order services, the total score of self-assessed research competence improved significantly after participation (mean difference = −2.091, 95% CI −3.401 to −0.781, *p* = 0.003). Subscale scores with significant improvements included literature review (mean difference = −0.424, 95% CI −0.756 to −0.092, *p* = 0.014), research design (mean difference = −0.606, 95% CI −1.122 to −0.090, *p* = 0.023), research practice (mean difference = −0.424, 95% CI −0.840 to −0.008, *p* = 0.046), and manuscript writing (mean difference = −0.636, 95% CI −1.104 to −0.169, *p* = 0.009). Total research self-efficacy scores and the results presentation dimension also improved significantly (mean difference = −0.242, 95% CI −0.420 to −0.064, p = 0.009; results presentation mean difference = −0.121, 95% CI −0.239 to −0.004, *p* = 0.044). Additionally, the total score of perceived organizational climate for innovation increased among postgraduate students (mean difference = −0.576, 95% CI −0.931 to −0.221, *p* = 0.002), mainly driven by improvements in resource provision (mean difference = −0.212, 95% CI −0.405 to −0.019, *p* = 0.033).

**Conclusion:**

The nursing research order service model, based on the “demand–resource” linkage, appears to enhance clinical nurses’ research capacity and self-efficacy, while showing observed improvements in research abilities, self-efficacy, and perception of the organizational research climate among nursing postgraduate students. This model may be particularly relevant for clinical settings with limited nursing research resources and could help cultivate a supportive research environment, promote the development of nurses’ research capabilities, and contribute to the advancement of the nursing discipline.

## 1 Introduction

Nursing research competence is defined as the ability to conduct research activities in a sustainable manner within a specific context (1). With the continuous development of the nursing discipline, research competence has become a key driver of disciplinary advancement (2). Enhancing the research capacity of healthcare professionals is also widely recognized as an important mechanism for improving the quality of health services and ensuring the efficient operation of healthcare systems (3). In the United States, an increasing number of nurse scientists embedded in healthcare organizations are engaged in generating new knowledge and applying research findings to support evidence-based practice, thereby improving nursing quality and patient outcomes (4). These nurse researchers play an increasingly significant role in advancing nursing theory and promoting the translation of evidence into clinical practice, and they are gradually receiving greater organizational support (4).

However, the academic career pathways and evidence-based practice capabilities of nurses remain underdeveloped (3). Hagan et al. (5) reported that clinical nurses have limited opportunities to engage in research. Another study found that more than 50% of nurses rated their personal research competence and research culture as low (6). A national survey in China similarly revealed that 60.90% of nurses had low levels of research competence (7). For clinical nurses, heavy workloads often constrain the time available for research (8, 9), while the lack of colleagues with research experience and the need to review a high volume of literature remain major barriers to research engagement (10). Improving nurses’ research self-efficacy, cultivating a supportive research environment, and enhancing the accessibility of research resources are all conducive to strengthening nursing research capacity. Evidence suggests that research self-efficacy is a key psychological factor influencing sustained research involvement; higher levels of self-efficacy not only foster research interest but also contribute to the production of higher-quality research outputs (7). Moreover, a positive research environment can stimulate nurses’ interest in research, enhance their competency, and promote the overall development of nursing teams (11). One study further indicated that the availability of research resources and the positivity of the research climate are strongly associated with individuals’ research participation and innovation (12).

According to the Resource-Based View (RBV), an organization’s competitive advantage derives from the acquisition, integration, and allocation of heterogeneous internal resources. Resources that are valuable, rare, inimitable, and non-substitutable are most likely to generate core competitiveness (13). In practice, graduate nursing students possess systematic theoretical knowledge and research skills but are often disconnected from clinical practice, whereas clinical nurses have rich specialty-specific experience but limited research competence. Using RBV as a conceptual framework, our hospital developed a nursing research order-service model based on a “demand–resource” linkage mechanism (hereafter referred to as the order-service model). The model aims to strategically acquire, integrate, and allocate the valuable research-related resources of both graduate students and clinical nurses through organizational intervention, foster a supportive research environment, and provide practical insights for enhancing overall clinical nursing research capacity.

## 2 Methods

### 2.1 Study design

A single-group, self-controlled before-and-after study was conducted to evaluate the effects of the nursing research order-service model on clinical nurses’ and graduate nursing students’ research competence, research self-efficacy, and perceived organizational innovation climate. This design allowed assessment of within-participant changes before and after participation in the intervention.

### 2.2 Participants

Participants included two groups—clinical nurses and graduate nursing students—who engaged in the nursing research order-service model from May to July 2025. The inclusion criteria were: (1) each clinical nurse was paired “one-to-one” with a graduate nursing student, and both parties participated in the entire service process; (2) both completed the assigned tasks within the scheduled timeline.

Exclusion criteria were: (1) clinical nurses or graduate students who joined the service process midway; (2) pairs in which interpersonal conflicts or coordination issues occurred.

### 2.3 Sample size

No *a priori* sample size calculation was performed, as this study adopted a real-world, practice-based design. All graduate students and clinical nurses who participated in the research demand–resource linkage program during the study period were consecutively included and invited to complete the questionnaire survey. Accordingly, the sample size was determined by actual participation in the program rather than by predefined recruitment targets.

### 2.4 Intervention

The nursing research order-service model facilitates efficient matching between the research needs of clinical nurses and the research services provided by graduate nursing students through the hospital’s Office Automation (OA) system. The implementation process consists of four main steps: order initiation, order evaluation and allocation, order acceptance and service delivery, and order evaluation and incentives.

Clinical nurses can submit service requests at any time via their personal OA system portal based on their research needs, such as research topic guidance, data analysis, or manuscript polishing. Upon receiving an order, the Nursing Department first conducts a preliminary assessment of the feasibility and innovativeness of the research request, and then matches it with a graduate nursing student whose research direction aligns with the request. After receiving the order, the graduate student conducts a comprehensive assessment of both the research request and their own research capacity. Upon confirmation of acceptance, a “one-to-one” research service is initiated. If the student declines the order, the Nursing Department reassigns it to another student.

The form of service is determined through consultation between the clinical nurse and the graduate student, and can be either guidance-based or participation-based. Guidance-based service primarily involves telephone communication, written feedback, online meetings, or face-to-face interactions. For this type of service, the Nursing Department provides both an order completion fee and a performance-based reward. Participation-based service involves collaborative research between the clinical nurse and the graduate student, with shared research outcomes; in this case, the Nursing Department provides only the order completion fee. Upon completion of the service, the applicant evaluates the service via the OA system portal. The Nursing Department regularly tracks and supervises service quality, and the order completion fees and performance rewards are disbursed every 6 months. For detailed information on the intervention, please refer to Table 1.

**Table 1:** Description of the nursing research order-service model using the TiDIER framework.

TiDIER item	Description
1. Name	Nursing research order-service model
2. Why (rationale, theory, goal)	To improve clinical nurses’ research competence and self-efficacy, and facilitate translation of clinical problems into research practice.
3. What (materials)	Hospital OA office system for order submission, evaluation, and tracking; structured forms for research requests; reward system for completed orders.
4. What (procedures)	1. Order initiation by clinical nurse via OA system (e.g., research topic guidance, data analysis, manuscript polishing). 2. Nursing Department evaluates feasibility and innovativeness, and matches graduate student. 3. Graduate student assesses request and own capacity; confirms acceptance. 4. “One-to-one” guidance begins; if declined, Nursing Department rematches. 5. Guidance can be “consultative” (phone, written feedback, online, face-to-face) or “participatory” (joint research with shared outcomes). 6. Completion evaluation by clinical nurse in OA system. 7. Nursing Department tracks service quality; fees and rewards disbursed every 6 months.
5. Who provided	Graduate nursing students, guided and supervised by Nursing Department staff.
6. How (delivery)	Individualized “one-to-one” guidance; delivery modes include phone, written feedback, online meetings, or in-person interactions; participatory collaboration if needed.
7. Where	Conducted within hospital OA system; meetings and communications online, by phone, or in designated hospital areas.
8. When and how much	Conducted during the service period (May–July 2025); task duration depends on complexity but is generally short (e.g., statistical analysis, topic guidance, manuscript polishing).
9. Tailoring (adaptations)	Service mode (guidance vs. participation) and communication method tailored to research request and participant preferences.
10. How well (planned)	Nursing Department monitored service process, tracked order completion, and ensured tasks were completed within scheduled timelines.
11. How well (actual)	All accepted orders completed by graduate students; initial mismatches promptly reassigned to maintain timeline and quality.
12. Modifications	No major modifications to the model. If initial pairing was unsuitable due to capability mismatch, graduate student reassigned promptly.

### 2.5 Outcome measures

(1) Demographic questionnaire

A self-designed questionnaire developed by the research team was used to collect demographic and professional information, including gender, age, professional title, education level, marital status, prior publication experience, and experience in research project applications.

(2) Self-assessed research competence scale.

The scale, revised by Pan et al. (14), includes 30 items across six dimensions: problem identification, literature review, research design, research practice, data handling, and manuscript writing. Items are rated on a 5-point Likert scale, with a total score ranging from 0 to 120. Higher scores indicate stronger self-perceived research competence. The internal consistency of the scale was reported as Cronbach’s *α* = 0.861.

(3) Research self-efficacy questionnaire.

Developed by Sun et al. (15), this questionnaire comprises 21 items across four dimensions: idea generation, problem formulation, research implementation, and results presentation. Responses are scored on a 5-point Likert scale, yielding a total score ranging from 21 to 105. Higher scores reflect greater self-efficacy in conducting research activities. The questionnaire demonstrated excellent reliability with Cronbach’s *α* = 0.958.

(4) Nurse organizational innovation climate scale.

Compiled by Qian et al. (16), this scale includes 21 items across three dimensions: organizational innovation incentives, resource provision, and management practices. Items are rated on a 5-point Likert scale, with total scores ranging from 21 to 105. Higher scores indicate a more positive perception of the organizational innovation climate. The overall Cronbach’s *α* of the scale was 0.938.

(5) Timing and administration of assessments.

For clinical nurses, assessments were conducted before the initiation and after the completion of the research order-service process. For graduate nursing students, assessments were conducted in May and July 2025. Questionnaires were administered online via the Wenjuanxing platform, and two staff members from the Nursing Department were responsible for the full process to ensure data completeness and timeliness.

### 2.6 Quality control

During the study, all survey personnel received standardized training to ensure proper questionnaire completion and consistent data collection. Data entry was performed with double-checking, and datasets were cleaned and verified to ensure accuracy.

### 2.7 Ethics statement

This study was approved by the Ethics Committee of Mianyang Central Hospital (Approval No: S20250375-02). All participants provided informed consent and voluntarily participated in the study.

### 2.8 Statistical analysis

Data were analyzed using SPSS version 25.0. Continuous variables were presented as mean ± standard deviation (SD). Within-group comparisons of clinical nurses and graduate nursing students before and after participation in the nursing research order-service model were conducted using paired-sample *t*-tests. For each outcome, the mean difference (post-intervention minus pre-intervention) and its 95% confidence interval (CI) were calculated to quantify the magnitude of change. Categorical variables were presented as frequencies and percentages and compared using the chi-square (*χ*^2^) test. All statistical tests were two-tailed, and a *p* < 0.05 was considered statistically significant.

## 3 Results

### 3.1 Research service orders and participant demographics

A total of 136 research service orders from clinical nurses were received. After an initial evaluation by the Nursing Department, 7 orders were rejected due to insufficient scientific validity or lack of innovation, while the remaining 129 orders were completed, yielding a completion rate of 94.85%. During the service process, 36 manuscripts and 25 research projects were completed. A total of 126 clinical nurses and 34 graduate nursing students participated in the nursing research order-service model. Among the 126 clinical nurses, 5.56% were male and 94.44% were female; 29.37% were aged 20–30 years, 59.52% were 31–40 years, and 11.11% were 41–50 years; 8.73% held an associate degree and 91.27% held a bachelor’s degree; the majority had 11–15 years of work experience (31.75%), and most held the title of senior nurse (60.33%). Among the 34 graduate nursing students, 2.94% were male and 97.06% were female; 29.41% were aged 20–30 years, 52.94% were 31–40 years, and 17.65% were 41–50 years; 29.41% had 6–10 years of work experience, and half held the title of senior nurse (50.00%). Detailed information is presented in Table 2.

**Table 2:** Demographic characteristics of clinical nurses and graduate nursing students.

Characteristic	Clinical nurses (*n* = 126)	Graduate nursing students (*n* = 34)
Gender		
Female	119 (94.44)	33 (97.06)
Male	7 (5.56)	1 (2.94)
Age		
20–30	37 (29.37)	10 (29.41)
31–40	75 (59.52)	18 (52.94)
41–50	14 (11.11)	6 (17.65)
Marital status		
Single	24 (19.05)	8 (23.53)
Married	97 (76.98)	25 (73.53)
Divorced or widowed	5 (3.97)	1 (2.94)
Number of children		
0	35 (27.78)	10 (29.41)
1	56 (44.44)	14 (41.18)
2	34 (26.98)	10 (29.41)
3	1 (0.80)	0 (0.00)
Childcare time outside work		
≤20%	16 (17.58)	10 (33.33)
21%–40%	18 (19.78)	6 (25.00)
41%–60%	18 (19.78)	5 (20.83)
61%–80%	24 (26.38)	1 (4.17)
81%–100%	15 (16.48)	4 (16.67)
Education level		
College Diploma	11 (8.73)	0 (0.00)
Bachelor’s Degree	115 (91.27)	0 (0.00)
Master’s Degree	0 (0.00)	34 (100.00)
Years of work experience (year)		
0–5	28 (22.22)	9 (26.47)
6–10	32 (25.40)	10 (29.41)
11–15	40 (31.75)	8 (23.53)
16–20	13 (10.32)	2 (5.88)
21–25	9 (7.14)	2 (5.88)
26–30	4 (3.17)	3 (8.83)
Employment status		
Permanent	52 (41.27)	26 (76.47)
Contract	74 (58.73)	8 (23.53)
Professional title		
Nurse	12 (9.52)	3 (8.82)
Registered nurse	26 (20.63)	5 (14.71)
Charge nurse	76 (60.33)	17 (50.00)
Deputy chief nurse	12 (9.52)	4 (11.76)
Chief nurse	0 (0.00)	5 (14.71)
Average number of night shifts per month		
0–2	46 (36.52)	28 (82.36)
11–12	3 (2.38)	2 (5.88)
13–14	3 (2.38)	2 (5.88)
15–16	3 (2.38)	0 (0.00)
3–4	12 (9.52)	0 (0.00)
5–6	26 (20.63)	0 (0.00)
7–8	28 (22.22)	0 (0.00)
9–10	5 (3.97)	2 (5.88)

### 3.2 Pre- and post-intervention comparison of clinical nurses’ self-assessed research competence, research self-efficacy, and perceived organizational innovation climate

Among the 126 nurses who submitted research orders, the total score of self-assessed research competence significantly improved after participation (mean difference = −0.611, 95% CI −0.931 to −0.291, *p* < 0.001). Significant improvements were also observed in several subscales: problem identification (mean difference = −0.079, 95% CI −0.148 to −0.010, *p* = 0.025), literature review (mean difference = −0.087, 95% CI −0.150 to −0.024, *p* = 0.007), research design (mean difference = −0.095, 95% CI −0.186 to −0.005, *p* = 0.039), research practice (mean difference = −0.079, 95% CI −0.145 to −0.014, *p* = 0.018), and data processing ability (mean difference = −0.175, 95% CI −0.277 to −0.072, *p* = 0.001). The problem formulation dimension of research self-efficacy also showed a significant increase (mean difference = −0.079, 95% CI −0.145 to −0.014, *p* = 0.018). However, no significant differences were observed in the manuscript writing dimension of the self-assessed research competence scale, the total score of the organizational innovation climate scale and its individual dimensions, or the total score and the dimensions of idea generation, research implementation, and results presentation in the research self-efficacy questionnaire (*p* > 0.05). Detailed results are presented in Table 3.

**Table 3:** Pre- and post-intervention scores of self-assessed research competence, research self-efficacy, and perceived organizational innovation climate in clinical nurses.

Project	Pre	Post	Mean difference	SD	SEM	95%CI lower	95%CI upper	*t*	*P*
Self-assessed research competence	52.29 ± 23.81	52.90 ± 23.02	−0.611	1.815	0.162	−0.931	−0.291	−3.779	<0.001
Problem identification	7.10 ± 2.40	7.18 ± 2.36	−0.079	0.392	0.035	−0.148	−0.010	−2.273	0.025
Literature review	11.32 ± 3.86	11.40 ± 3.80	−0.087	0.358	0.032	−0.15	−0.024	−2.736	0.007
Research design	7.37 ± 4.52	7.46 ± 4.40	−0.095	0.513	0.046	−0.186	−0.005	−2.085	0.039
Research practice	9.53 ± 5.58	9.61 ± 5.47	−0.079	0.371	0.033	−0.145	−0.014	−2.401	0.018
Data processing	6.08 ± 4.66	6.25 ± 4.44	−0.175	0.581	0.052	−0.277	−0.072	−3.375	0.001
Manuscript writing	10.90 ± 6.04	10.99 ± 5.77	−0.095	0.871	0.078	−0.249	0.058	−1.227	0.222
Research self-efficacy	64.56 ± 21.04	64.60 ± 20.76	−0.048	0.979	0.087	−0.220	0.125	−0.546	0.586
Conception generation	17.14 ± 5.32	17.11 ± 5.31	0.032	0.251	0.022	−0.013	0.076	1.420	0.158
Problem formulation	12.46 ± 4.41	12.54 ± 4.28	−0.079	0.371	0.033	−0.145	−0.014	−2.401	0.018
Research implementation	20.48 ± 7.41	20.45 ± 7.35	0.032	0.356	0.032	−0.031	0.095	1.000	0.319
Results presentation	14.47 ± 5.20	14.50 ± 5.11	−0.032	0.455	0.041	−0.112	0.048	−0.783	0.435
Organizational innovation climate	95.13 ± 10.49	95.12 ± 10.43	0.016	0.780	0.069	−0.122	0.153	0.229	0.820
Innovation incentive	32.53 ± 3.72	32.48 ± 3.73	0.056	0.478	0.043	−0.029	0.140	1.303	0.195
Resource provision	26.76 ± 3.23	26.78 ± 3.19	−0.016	0.506	0.045	−0.105	0.073	−0.352	0.725
Management practices	35.84 ± 4.61	35.87 ± 4.59	−0.024	0.199	0.018	−0.059	0.011	−1.346	0.181

### 3.3 Pre- and post-intervention comparison of graduate nursing students’ self-assessed research competence, research self-efficacy, and perceived organizational innovation climate

Among the 34 postgraduate nursing students who provided research order services, the total score of self-assessed research competence improved significantly after participation (mean difference = −2.091, 95% CI −3.401 to −0.781, *p* = 0.003). Subscale scores with significant improvements included literature review (mean difference = −0.424, 95% CI −0.756 to −0.092, *p* = 0.014), research design (mean difference = −0.606, 95% CI −1.122 to −0.090, *p* = 0.023), research practice (mean difference = −0.424, 95% CI −0.840 to −0.008, *p* = 0.046), and manuscript writing (mean difference = −0.636, 95% CI −1.104 to −0.169, *p* = 0.009). Total research self-efficacy scores and the results presentation dimension also improved significantly (mean difference = −0.242, 95% CI −0.420 to −0.064, *p* = 0.009; results presentation mean difference = −0.121, 95% CI −0.239 to −0.004, *p* = 0.044). Additionally, the total score of perceived organizational climate for innovation increased among postgraduate students (mean difference = −0.576, 95% CI −0.931 to −0.221, *p* = 0.002), mainly driven by improvements in resource provision (mean difference = −0.212, 95% CI −0.405 to −0.019, *p* = 0.033). However, no significant differences were observed in the scores for the problem identification and data handling dimensions of the self-assessed research competence scale, the innovation incentives and management practices dimensions of the organizational innovation climate scale, or the idea generation, problem formulation, and research implementation dimensions of the research self-efficacy questionnaire (*p* > 0.05). Detailed results are presented in Table 4.

**Table 4:** Pre- and post-intervention scores of self-assessed research competence, research self-efficacy, and perceived organizational innovation climate in graduate nursing students.

Project	Pre	Post	Mean difference	SD	SEM	95% CI lower	95% CI upper	*t*	*p*
Self-assessed research competence	84.18 ± 20.03	86.27 ± 17.10	−2.091	3.694	0.643	−3.401	−0.781	−3.251	0.003
Problem identification	8.27 ± 2.08	8.36 ± 2.10	−0.091	0.459	0.08	−0.253	0.072	−1.139	0.263
Literature review	15.33 ± 3.42	15.76 ± 3.05	−0.424	0.936	0.163	−0.756	−0.092	−2.603	0.014
Research design	13.24 ± 4.26	13.85 ± 3.38	−0.606	1.456	0.254	−1.122	−0.090	−2.390	0.023
Research practice	16.64 ± 4.33	17.06 ± 3.91	−0.424	1.173	0.204	−0.840	−0.008	−2.077	0.046
Data processing	12.00 ± 4.30	12.03 ± 3.26	−0.030	0.918	0.16	−0.356	0.295	−0.190	0.851
Manuscript writing	18.56 ± 4.63	19.24 ± 3.64	−0.636	1.319	0.23	−1.104	−0.169	−2.772	0.009
Research self-efficacy	83.85 ± 14.20	84.09 ± 14.28	−0.242	0.502	0.087	−0.420	−0.064	−2.775	0.009
Conception generation	20.70 ± 3.52	20.76 ± 3.61	−0.061	0.242	0.042	−0.147	0.025	−1.437	0.160
Problem formulation	15.79 ± 3.11	15.97 ± 3.08	−0.182	0.584	0.102	−0.389	0.025	−1.789	0.083
Research implementation	27.18 ± 5.27	27.21 ± 5.27	−0.030	0.174	0.030	−0.092	0.031	−1.000	0.325
Results presentation	20.03 ± 3.28	20.15 ± 3.29	−0.121	0.331	0.058	−0.239	−0.004	−2.101	0.044
Organizational innovation climate	91.27 ± 10.32	91.85 ± 9.89	−0.576	1.001	0.174	−0.931	−0.221	−3.304	0.002
Innovation incentive	31.76 ± 3.64	31.91 ± 3.37	−0.152	0.619	0.108	−0.371	0.068	−1.407	0.169
Resource provision	25.52 ± 3.05	25.73 ± 2.91	−0.212	0.545	0.095	−0.405	−0.019	−2.235	0.033
Management practices	34.00 ± 5.25	34.21 ± 5.18	−0.212	0.740	0.129	−0.474	0.050	−1.647	0.109

## 4 Discussion

The implementation of the nursing research order-service model effectively facilitated bidirectional empowerment between clinical nurses and graduate nursing students, demonstrating the practical feasibility and value of the “demand–resource” linkage mechanism (17). All research service orders were initiated by clinical nurses. Through the “one-to-one” research service, clinical nurses exhibited significant improvements in research-related competence and research self-efficacy, indicating that the model can meet individualized research needs and assist nurses in translating clinical problems into research practice. Meanwhile, graduate nursing students participating as service providers also showed enhanced research competence, research self-efficacy, and perception of organizational innovation climate, suggesting that the model benefits not only clinical nurses with limited research experience but also graduate students with an existing research foundation. This finding highlights the bidirectional empowerment characteristic of the model and provides a reference for future exploration of integrating research services with clinical practice.

### 4.1 Effectiveness in enhancing clinical nurses’ research-related competence

Research competence is a core ability for clinical nurses to transition from “problem observers” to “research facilitators,” playing a crucial role in promoting evidence-based practice and advancing professional development. However, clinical nurses generally exhibit limited research competence due to insufficient professional training, uneven resource distribution, and heavy workloads. Within the research order-service model, the “one-to-one” service effectively met the individualized research needs of clinical nurses. Under the systematic guidance, practical training, and collaborative division of labor provided by graduate nursing students, multiple dimensions of self-assessed research competence showed significant improvement. Specifically, clinical nurses demonstrated statistically significant differences in key competence dimensions, including problem identification, literature review, research design, research implementation, and data handling, suggesting a positive impact of the model on enhancing research competence. Although the observation period was short and formal outcomes such as manuscript publication rates and successful project approvals were not tracked, the completion of 36 manuscripts and 25 research projects partially reflects the model’s facilitation of research output. This approach, characterized by “learning through demand” and “training through practice,” can, to some extent, shorten the time from clinical problem identification to research outcomes and improve nursing research efficiency.

Research self-efficacy is a core psychological factor influencing individuals’ willingness to participate in research and their research output, referring to one’s confidence in the ability to complete specific research tasks (7, 18). High levels of research self-efficacy can effectively stimulate nurses’ interest in research and enhance their initiative and intrinsic motivation to engage in sustained research activities. Studies have shown that encouraging clinical nurses with research or evidence-based experience to participate in programs such as “research boot camps” can improve their research competence and confidence within an interdisciplinary collaborative environment. This is achieved through systematic training focused on problem formulation, literature retrieval, data management, and statistical analysis (4). The nursing research order-service model exerts a similar facilitative effect. In this study, the score for the problem formulation dimension of research self-efficacy increased significantly, indicating that clinical nurses participating in this model gradually developed the awareness and ability to transform clinical practice problems into research questions. This finding is consistent with previous research showing that in-service nurses generally have higher confidence in literature retrieval and database usage, but are relatively weaker in critical appraisal and research evaluation (18).

### 4.2 Effectiveness in enhancing graduate nursing students’ research-related competence

For graduate nursing students serving as research service providers, their overall self-assessed research competence score increased after participation, particularly in key competence dimensions such as literature review, research design, research practice, and manuscript writing, all showing significant improvement. These results indicate that the model is not only effective for clinical nurses with relatively limited research experience but also facilitates competence enhancement in graduate nursing students with an existing research foundation, highlighting the bidirectional empowerment feature of the model.

Meanwhile, the total score of research self-efficacy among graduate nursing students showed a notable increase, especially in the results presentation dimension, suggesting that this service model strengthened their abilities in data analysis and research output communication. Research self-efficacy is a key psychological factor influencing individuals’ sustained engagement in research activities (7). In research practice, results presentation not only reflects the accurate expression of statistical findings but also demonstrates the researcher’s ability to synthesize data and integrate graphical and textual information. Improvement in this dimension indicates that graduate nursing students enhanced their competence in critical steps such as data interpretation, research logic construction, and dissemination of findings.

In addition, graduate nursing students’ perceptions of the organizational research innovation climate were significantly enhanced, including the total score of the organizational innovation climate scale and the resource provision dimension. This indicates that while the model promoted individual research competence, it also optimized students’ subjective perception of the research support system. Perceived research innovation climate and resource availability are important external factors influencing sustained engagement in research. Improvement in these areas can stimulate higher levels of motivation and creativity among graduate nursing students (12). Previous studies have emphasized that research climate and resource perception do not exist in isolation; they are shaped by multiple interacting factors, including enhancement of research competence, activation of research motivation, availability of material and institutional resources, collaboration between academic and clinical settings, and strong leadership support (12, 19). Leaders, as key drivers of change in the research environment, not only strengthen the organizational research climate through policy design and cultural guidance but also provide a stable and sustainable foundation for continuous research participation by fostering trust-based team relationships (12).

### 4.3 Limitations and potential extensions of the nursing research order-service model

At present, the nursing research order-service model has shown preliminary effectiveness in enhancing clinical nurses’ research engagement and facilitating the translation of clinical problems into research practice. To further maximize its practical value, nursing research objectives were integrated into the implementation of new nursing techniques and projects, promoting early integration of nursing innovation and research. However, several limitations remain in practical application. First, the research requests submitted by clinical nurses often lack precision, such as vague definitions or unclear research objectives, requiring substantial effort from graduate nursing students to assist in clarifying the requests. Second, during the actual research guidance and collaborative process, mismatched schedules between graduate students and clinical nurses due to workload constraints can affect the time invested and the effectiveness of the service. Third, the 126 participating clinical nurses were unevenly distributed across departments; departments with a strong research culture were able to effectively utilize research resources, whereas participation from other departments was relatively low. Most departments contributed only a few participants, and due to this dispersed and uneven distribution, conducting a formal statistical test for potential interaction effects between departmental research culture and primary outcomes would be challenging and might yield unreliable results. This limitation has been acknowledged here to transparently reflect the potential influence of departmental distribution on the study findings. Finally, the current service providers are limited to graduate nursing students, and the scope of service is narrow, making it difficult to meet the diverse and higher-level research needs of clinical nurses, such as those related to ethics, high-level manuscripts, or complex research projects. These challenges need to be continuously addressed and optimized as the model develops.

In addition, there are several methodological limitations to this study. A self-controlled before–after design without a parallel control group was employed, which limits the ability to draw causal inferences. The observed improvements may have been influenced by confounding factors, such as concurrent training activities, maturation effects—including potential improvements in graduate nursing students’ research competence over time due to their ongoing graduate studies—or participation-related effects—rather than being solely attributable to the intervention model. The sample size was determined by the actual number of participants in the program, rather than by *a priori* calculation, reflecting the real-world, practice-based nature of the study. Moreover, the intervention period was relatively short, which may have constrained the magnitude of observable changes. Potential self-selection bias may also limit the generalizability of the findings. Regarding participant inclusion, no pairs were excluded due to interpersonal conflicts. For instances in which the initial pairing of clinical nurses and graduate nursing students was not optimal, the graduate student was promptly reassigned to ensure timely completion of the research task. Although these adjustments did not constitute formal exclusions, they may introduce minor potential bias, which should be considered when interpreting the results. These methodological considerations indicate that the results should be interpreted as preliminary and exploratory, and that future studies employing controlled or longitudinal designs are warranted to verify causal effects and evaluate longer-term outcomes.

Potential improvements include systematically identifying the true research needs of clinical departments and nurses, categorizing resource bottlenecks in research topic selection, design, implementation, and outcome translation; developing a modular course library covering the entire research process to support foundational research guidance; and establishing multidisciplinary research support teams, including experts in ethics, statistics, epidemiology, public health, and information science.

## 5 Conclusion

The nursing research order-service model, based on the “demand–resource” linkage, utilizes organizational interventions to acquire, integrate, and allocate research resources from both graduate nursing students and clinical nurses. This model effectively supports clinical nurses in addressing research needs and enhances their research-related competence and self-efficacy. Meanwhile, graduate nursing students also benefit in terms of competence and perception of the research climate, highlighting the bidirectional empowerment characteristic of the model. These findings suggest that the research order-service model has practical value in facilitating research engagement and translating clinical problems into research practice, and further studies are warranted to evaluate its broader applicability and long-term outcomes.

## Data Availability

The raw data supporting the conclusions of this article will be made available by the authors, without undue reservation.
